# Large-scale statistical analysis of *Mycobacterium tuberculosis* genome sequences identifies compensatory mutations associated with multi-drug resistance

**DOI:** 10.1038/s41598-024-62946-8

**Published:** 2024-05-29

**Authors:** Nina Billows, Jody Phelan, Dong Xia, Yonghong Peng, Taane G. Clark, Yu-Mei Chang

**Affiliations:** 1 Royal Veterinary College, University of London, London, UK; 2 Faculty of Infectious and Tropical Diseases, London School of Hygiene and Tropical Medicine, London, UK; 3 Manchester Metropolitan University, Manchester, UK; 4 Faculty of Epidemiology and Population Health, London School of Hygiene and Tropical Medicine, London, UK

**Keywords:** Evolutionary biology, Genomics, Computational biology and bioinformatics, Bacterial genomics, Antimicrobial resistance

## Abstract

Tuberculosis (TB), caused by *Mycobacterium tuberculosis*, has a significant impact on global health worldwide. The development of multi-drug resistant strains that are resistant to the first-line drugs isoniazid and rifampicin threatens public health security. Rifampicin and isoniazid resistance are largely underpinned by mutations in *rpoB* and *katG* respectively and are associated with fitness costs. Compensatory mutations are considered to alleviate these fitness costs and have been observed in *rpoC/rpoA* (rifampicin) and *oxyR’-ahpC* (isoniazid). We developed a framework (CompMut-TB) to detect compensatory mutations from whole genome sequences from a large dataset comprised of 18,396 *M. tuberculosis* samples. We performed association analysis (Fisher’s exact tests) to identify pairs of mutations that are associated with drug-resistance, followed by mediation analysis to identify complementary or full mediators of drug-resistance. The analyses revealed several potential mutations in *rpoC* (N = 47), *rpoA* (N = 4), and *oxyR’-ahpC* (N = 7) that were considered either ‘highly likely’ or ‘likely’ to confer compensatory effects on drug-resistance, including mutations that have previously been reported and validated. Overall, we have developed the CompMut-TB framework which can assist with identifying compensatory mutations which is important for more precise genome-based profiling of drug-resistant TB strains and to further understanding of the evolutionary mechanisms that underpin drug-resistance.

## Introduction

Tuberculosis (TB) is a leading cause of mortality and morbidity worldwide^1^. TB is caused by members of the *Mycobacterium tuberculosis* complex (MTBC) and has traditionally been treated using a long course of antimicrobial drugs, including the first-line drugs such as rifampicin and isoniazid. Multidrug resistant TB (MDR-TB) strains are resistant to isoniazid (HR-TB) and rifampicin (RR-TB) and present a major challenge to public health and TB prevention globally^1^. RR-TB is mostly underpinned by mutations in *rpoB,* whilst HR-TB is caused by mutations in *inhA* and *katG.* However, the mechanisms that underpin drug-resistance are complex and the full repertoire of mutations involved remains unknown.

Rifampicin inhibits translation by targeting the RNA polymerase β-subunit *(rpoB*). In contrast, isoniazid is a prodrug that is oxidised by catalase peroxidase (*katG*), a protein involved in response to oxidative stress, to produce the pharmacologically active form of the drug^2^. Although mutations in both *rpoB* and *katG* can contribute to drug-resistance, they may also impair the biological function of the proteins they encode, leading to a reduction in bacterial fitness^3,4^. Mutations in *rpoB* may reduce the efficiency of translation^5^. In addition, *katG* mutations may lead to reduced virulence through reducing the ability for the bacterium to survive in the macrophage and withstand the host immune response due to oxidative stress^6^. This may have wider impacts on bacterial fitness, including transmission and growth^7–10^. Mutations in *rpoB* and *katG* have been observed at high frequency and have been demonstrated to have varying effects on bacterial fitness. For example, early in vitro experiments showed that the S450L *rpoB* mutation, the most common rifampicin-resistance mutation, had relatively minimal impact on fitness^5^. This may be due to the development of secondary mutations that compensate for the fitness costs associated with drug-resistance.

Compensatory mutations can mitigate the fitness costs of drug-resistance through altering the structure of the drug-target or by performing a similar function. Compensatory mutations have therefore been proposed in *rpoA* and *rpoC* which encode the α and β’-subunits of RNA polymerase respectively. High confidence compensatory mutations were previously reported by Comas et al*.,* 2012 and underwent validation to confirm their impact on bacterial fitness^5^. Additionally, secondary mutations have also been observed in the *oxyR’-ahpC* promoter region that increase the expression of AhpC (Alkyl hydroperoxide reductase C)^11^. AhpC is typically undetectable in sensitive strains of *M. tuberculosis* but is known to play a role in cell protection against oxidative stress in other bacterial species^12^. Overexpression of AhpC can help isoniazid-resistant *M. tuberculosis* isolates to overcome the impact of *katG* mutations on survival caused by reduced activity of catalase peroxidase (KatG) which also plays a role in response to oxidative stress^11^. Therefore, *oxyR’-ahpC* mutations are proposed to have compensatory effects on the evolution of isoniazid resistance, but are not a direct cause^13,14^. This is also considered to explain the reduced sensitivity to isoniazid in *Mycobacterium smegmatis,* where o*xyR’-ahpC* mutations drive AhpC expression^6,15^. More recently, whole genome sequencing (WGS) has assisted the discovery of compensatory mutations in *rpoA, rpoC* and *oxyR’-ahpC* using comparative genomics and traditional association tests, such as genome wide association studies (GWAS) and Fisher’s exact tests^7,16–32^. However, such analyses do not consider the interactions that occur between mutations to contribute to drug-resistance. Additionally, many previous studies have been performed with small sample sizes.

We developed a methodological framework (CompMut-TB) and leveraged large-scale *M. tuberculosis* WGS and phenotypic drug susceptibility test (pDST) data to detect compensatory mutations. CompMut-TB is based on association and mediation analyses. In general, mediation analysis is used to explore the process by which a mediator variable (M) affects the relationship between the independent variable (X) and the outcome variable (Y), the significance of which is determined by the indirect effect. This indirect effect represents the part of the relationship between X and Y that is mediated through M and considers the influence of compensatory mutations (M) on the relationship between the drug-resistance mutation (X) and the drug-resistant phenotype (Y). We apply our framework to target regions (*rpoA, rpoC* and *oxyR’-ahpC*) known to confer compensatory effects and detect potential compensatory, non-synonymous mutations in *rpoA* and *rpoC* and intergenic mutations in *oxyR’-ahpC*, some of which have been previously validated. The identification of compensatory mutations is important to broaden understanding of drug-resistance mechanisms and support genome-based profiling of drug-resistant strains.

## Results

### *M. tuberculosis* genetic diversity and drug-resistant phenotypes

This study utilised WGS and pDST data available in the public domain which have previously undergone expert curation. Data for 18,396 M*. tuberculosis* isolates were analysed in this study. After removing isolates with missing rifampicin and isoniazid pDST results, 18,088 isolates and 17,895 isolates remained for subsequent analysis respectively. *M. tuberculosis* isolates were globally distributed, and the major lineages were represented, including L1-L7 and zoonotic lineages (Table 1)**.** Principal component analysis revealed isolates clustered by lineage and most isolates belonged to the modern MTBC lineages (L4, 48.2%; L2, 25.0%; L3, 15.4%) (Supplementary Figure S1). Approximately 29% isolates were rifampicin-resistant, 34% were isoniazid-resistant and 25% were resistant to both isoniazid and rifampicin (MDR-TB). A total of 945,771 SNPs and 102,617 Indels were called across WGS, in which the vast majority (~ 98%) had low minor allele frequency (< 1%). A considerable number of non-synonymous mutations were observed in *rpoB* (N = 561), *rpoC* (N = 575), *katG* (N = 540), whilst fewer were identified in *rpoA* (N = 135) and *oxyR’-ahpC* (intergenic, N = 48). Most rifampicin-resistant isolates contained a non-synonymous mutation in *rpoB* (98%) and *rpoC* (67%), but only 5% contained a non-synonymous mutation in *rpoA* (Table 1). Likewise, most isoniazid resistant samples contained a non-synonymous mutation in *katG* (92%), but a smaller proportion contained a mutation in *oxyR’-ahpC* (16%). The distribution of non-synonymous mutations in *rpoA, rpoB,* and *rpoC* varied across lineages (Table 1). For example, 95% of rifampicin-resistant isolates in L4 contained a mutation in *rpoB*, in comparison to 100% in all other lineages. Additional mutations observed within these samples and their confidence grading according to the WHO mutation catalogue are also reported^33,34^. However, such mutations were observed at low frequency with ‘uncertain significance’ and were not strong candidates for potential drug-resistance mutations (Supplementary Table S1).

**Table 1:** Descriptive summary of the global TB dataset.

Lineage (L)	Total Number	Rifampicin-resistant TB (% of dataset)		Isoniazid-resistant TB (% of dataset)		Multidrug resistant TB		Number of Isolates with mutations in *rpoA* (% in RR-TB)*		Number of Isolates with mutations in *rpoB* (% in RR-TB)*		Number of Isolates with mutations in *rpoC* (% in RR-TB)*		Number of Isolates with mutations in *katG* (% in HR-TB)*		Number of Isolates with mutations in *oxyR'-ahpC* (% in HR-TB)*	
L1	1864	184	(10%)	340	(18%)	171	(9%)	63	(4%)	1864	(100%)	1864	(100%)	1864	(100%)	16	(1%)
L2	4605	2601	(56%)	2652	(58%)	2169	(47%)	146	(5%)	4605	(100%)	2050	(61%)	4590	(99%)	144	(5%)
L3	2833	489	(17%)	651	(23%)	463	(16%)	35	(3%)	2833	(100%)	518	(39%)	2827	(99%)	2833	(100%)
L4	8875	2121	(24%)	2533	(29%)	1797	(20%)	413	(6%)	3695	(95%)	5836	(77%)	2506	(82%)	206	(7%)
L5	31	2	(6%)	7	(23%)	2	(6%)	20	(0%)	31	(100%)	3	(50%)	31	(100%)	1	(14%)
L6	44	3	(7%)	6	(14%)	3	(7%)	18	(67%)	44	(100%)	13	(67%)	44	(100%)	4	(67%)
L7	2	0	(0%)	0	(0%)	0	(0%)	2	(–)	2	(–)	2	(–)	2	(–)	0	(–)
Zoonotic	142	3	(2%)	8	(6%)	3	(2%)	9	(0%)	142	(100%)	27	(100%)	142	(100%)	4	(0%)
Total	18,396	5403	(29%)	6197	(34%)	4608	(25%)	706	(5%)	13,216	(98%)	10,313	(67%)	12,006	(92%)	3208	(16%)

*non-synonymous mutation; *RR-TB* Rifampicin-resistant, *HR-TB* Isoniazid-resistant.

### Putative compensatory mutations in* oxyR’-ahpC*

We first applied the CompMut-TB framework to identify potential compensatory mutations in the *oxyR’-ahpC* region. Two *oxyR’-ahpC* mutations were identified as ‘highly likely’ compensatory mutations using our analysis framework (Table 2)**.** The *oxyR’-ahpC* -48G > A (2726145G > A; P = 1.3 × 10^−22^, Fisher’s exact test) and *katG* S315R (P = 4.2 × 10^−24^, Fisher’s exact test) mutations were associated with the isoniazid resistant phenotype. It was found that *oxyR’-ahpC* −48G > A was also a significant complementary mediator between *katG* S315R and isoniazid resistance (*ab* estimate = 0.09, 95% CI = 0.04–0.14, standardised estimate = 0.01) (Table 2)**.** The *ahpC -*48G > A variant was also highlighted as a ‘likely’ putative compensatory mutation for *katG* S315T, as well as two other mutations including *ahpC* -52C > A (2726141C > A) and *ahpC* − 47G > GT (2726146G > GT) (Supplementary Table S2). Whilst most potential compensatory mutations had a partial mediating effect on the relationship between a *katG* drug-resistance mutation and isoniazid resistance, *katG* Y413C was fully mediated by *ahpC* − 52C > T and contributed to approximately 48% of the total effect (Table 2**, **Supplementary Table S2). Apart from *ahpC* − 47G > GT, the compensatory effects of the ‘highly likely’ and ‘likely’ mutations identified using the CompMut-TB framework have previously been validated. All potential compensatory mutations in *oxyR’-ahpC* occurred across multiple MTBC lineages but were unevenly distributed amongst sub-lineages and were mostly observed in lineage 2.2.1 (Supplementary Table S3).

**Table 2:** ‘Highly likely’ and previously validated compensatory mutations identified using CompMut-TB.

Genomic Region	Potential Compensatory Mutation	Lineages (N)	Associated Drug-resistant Mutation (Adjusted P-value)	Associated Drug-resistant Phenotype (Adjusted P-value)	Indirect Effect, ab (95% CI)	Probability	Experimental Validation	High Confidence Mutation (Comas et al*.,* 2012)^5^
*oxyR'-ahpC*	-52C > T	2.2.1 (28), 2.2.1.1 (5), 4 (1), 4.1 (1), 4.1.1 (1), 4.1.2 (1), 4.1.2.1 (6), 4.2.1 (1), 4.3.2 (1), 4.3.3 (7), 4.3.4.2.1 (1), 4.4.2 (5), 4.5 (4), 4.6.2.2 (1), 4.7 (1), 4.9 (1), 6 (1)	*katG* Y413C (2.29E−04)	Isoniazid (1.03E−22)	2.48E-01 (6.97E− 02:4.94E-01)	Highly likely	No	No
*oxyR'-ahpC*	-48G > A	2.2.1 (26), 2.2.1 (3), 2.2.2 (1), 4 (1), 4.1.1 (1), 4.1.1.3 (15), 4.1.2.1 (1), 4.1.2.1 (2), 4.2.1 (4), 4.2.2 (1), 4.3.3 (1), 4.3.4.2 (1), 4.5 (4), 4.8 (1), 6 (2)	*katG* S315R (1.90E−21)	Isoniazid (7.78E−22)	9.20E-02 (4.38E−2:1.43E-01)	Highly likely	No	No
*rpoC*	V483A	1.2.1 (1), 2.2.1 (42), 2.2.1.1 (1), 2.2.2 (2), 3 (5), 3.1.1 (5), 3.1.2.1 (1), 4.1 (1), 4.1.1.2 (1), 4.1.1.3 (8), 4.1.2.1 (3), 4.2.1 (4), 4.3.2 (3), 4.3.3 (2), 4.3.4.1 (2), 4.3.4.2 (2), 4.3.4.2.1 (1), 4.5 (2), 4.8 (1), 4.8 (3)	*rpoB* Q432P (1.52E-02)	Rifampicin (7.64E−44)	1.09E-01 (2.34E−02:2.45E-01)	Highly likely	No	No
*rpoC*	W484G	1.1.2 (1), 2.2.1 (4), 2.2.1 (11), 2.2.2 (1), 2.2.2 (1), 3 (2), 3 (3), 3.1.2 (1), 3.1.2.1 (1), 4 (1), 4.2.2.1 (1), 4.4.1.1 (1), 4.6.2.2 (1), 4.8 (1), 4.8 (5)	*rpoB* V170F (4.92E-07)	Rifampicin (8.62E−18)	6.02E-02 (1.98E−02:1.03E-01)	Highly likely	No	No
*rpoC*	E1033A	2.2.1 (2), 2.2.1.1 (4), 4 (1), 4.1.1.3 (5), 4.1.2.1 (1), 4.3.3 (1), 4.4.1.1 (26), 4.8 (1)	*rpoB* I491F (4.17E-46)	Rifampicin (2.88E−19)	1.54E-01 (1.01E-01:2.13E−01)	Highly likely	No	No
*rpoC*	V483G	1.1.1 (2), 1.1.2 (2), 1.2.1 (1), 1.2.2 (2), 2.1 (1), 2.2.1 (99), 2.2.1.1 (57), 2.2.2 (55), 3 (33), 3.1.2 (2), 4 (3), 4.1 (1), 4.1.1.3 (1), 4.1.2.1 (1), 4.2.1 (17), 4.2.2 (5) 4.2.2.1 (1), 4.3.2 (2), 4.3.4.1 (3), 4.3.4.2 (7), 4.4 (2), 4.4.1.1 (3), 4.4.2 (1), 4.5 (10), 4.7 (6), 4.8 (2), 6 (2)	*rpoB* Q432P (3.84E−06)	Rifampicin (1.96E−152)	2.57E-01 (1.05E−01:4.32E−01)	Highly likely	Yes	Yes
			*rpoB* S450L (3.48E−198)	Rifampicin (1.96E−152)	2.74E-03 (6.55E−04:4.75E−03)	Likely	Yes	Yes
*rpoC*	I491V	1.1.1 (1), 1.1.2 (1), 1.2.1 (1), 2.2.1 (38), 2.2.1.1 (1), 3 (4), 3.1.1 (1), 4 (1), 4.1.1.3 (3), 4.2.1 (2), 4.2.2 (6), 4.3.3 (14), 4.3.4.2 (6), 4.3.4.2.1 (1), 4.4.2 (2), 4.5 (1)	*rpoB* S450L (1.30E−53)	Rifampicin (4.62E−42)	4.73E−04 (1.59E−04:1.01E−03)	Likely	Yes	Yes
*rpoC*	D485N	2.2.1 (20), 2.2.1.2 (6), 4.2.1 (12)	*rpoB* S450L (1.25E−24)	Rifampicin (8.89E−19)	1.01E−04 (5.91E−05:1.55E−04)	Likely	Yes	Yes
*rpoC*	L516P	1.2.1 (2), 2.2.1 (28), 3 (4), 4 (1), 4.1.2.1 (8), 4.2.2 (7), 4.3.3 (2), 4.3.4.1 (1), 4.7 (3), 4.8 (1)	*rpoB* S450L (2.04E−29)	Rifampicin (1.89E−26)	6.08E-04 (1.04E−04:1.26E−03)	Likely	Yes	Yes
*rpoC*	P434R	2.2.1 (4), 2.2.1.1 (4), 4.8 (1)	*rpoB* S450L (9.74E−05)	Rifampicin (4.47E−05)	2.50E−05 (9.83E−06:4.63E−05)	Likely	No	Yes
*rpoC*	I491T	2.2.1 (45), 3 (6), 4.1.2.1 (1), 4.4.1.1 (1)	*rpoB* S450L (1.01E−32)	Rifampicin (6.53E-27)	3.85E-04 (1.00E−04:9.92E-04)	Likely	No	Yes
*rpoC*	N698S	1.1.2 (1), 2.2.1 (102), 2.2.1.1 (2), 3 (2), 3.1.1 (1), 4.1.2.1 (1), 4.3.3 (3), 4.3.4.1 (1), 4.3.4.2 (1), 4.4.2 (1)	*rpoB* S450L (5.12E−76)	Rifampicin (2.93E−59)	5.73E−04 (2.25E−04:1.10E−03)	Likely	No	Yes

*CI* Confidence interval.

### Putative compensatory mutations in *rpoC* and *rpoA*

We also applied the CompMut-TB framework to identify potential compensatory mutations in *rpoC* and *rpoA.* Four *rpoC* mutations were ‘highly likely’ compensatory mutations according to the analysis framework (Table 2). Known drug-resistance mutation *rpoB* Q432P was associated with two missense mutations in *rpoC* that mediated its relationship with rifampicin resistance. This included *rpoC* V483G (*ab* estimate = 0.26, 95% CI = 0.11–0.43, standardised estimate = 0.02) and *rpoC* V483A (*ab* estimate = 0.11, 95% CI = 0.02–0.25, standardised estimate = 0.01) (Table 2). Two other complementary mediators were identified as ‘highly likely’ compensatory mutations in *rpoC*, including *rpoC* E1033A and *rpoC* W484G which were associated with known drug-resistance mutations *rpoB* I491F (P = 2.2 × 10^−49^, Fisher’s exact test) and V170F (P = 1.4 × 10^−9^, Fisher’s exact test) respectively (Table 2).

Furthermore, several mutations in *rpoC* and *rpoA* were regarded as ‘likely’ to be compensatory given their lower indirect effect size in comparison the putative compensatory mutations described above (Supplementary Table S2). Despite their lower effect size, many of the mutations have previously been reported to express compensatory effects (Table 2). A total of 45 *rpoC* mutations were categorised as ‘likely’ to be a compensatory mutation, 41 (91.1%) of which had previously been reported in the literature (Supplementary Table S2). All mutations were associated with and mediated the response for the most prevalent *rpoB* mutation S450L, except for *rpoC* S561P and L566V which were associated with *rpoB* H445R (Supplementary Table S2). Therefore, the lower indirect effect size of ‘likely’ compensatory mutations may in part be driven by the smaller loss in fitness that has previously been indicated for *rpoB* S450L^3^. One mutation, *rpoC* G332S occurred independently of any drug-resistance mutation. However, after further investigation *rpoC* G332S was found in isolates with lineage-specific mutations in *rpoB* which were removed prior to the analysis. Moreover, no ‘highly likely’ compensatory mechanisms were observed in *rpoA*. However, seven *rpoA* mutations (G31S, A180V, T181A, V183G, V183A, E184D and T187N) were significantly associated with a *rpoB* drug-resistance mutation and mediated the relationship with rifampicin resistance, all of which have been described previously (Supplementary Table S2). Potential compensatory mutations in *rpoC* and *rpoA* were observed across several sub-lineages and most isolates with a potential compensatory mutation in *rpoC* belonged to lineage 2.2.1 (Supplementary Table S3).

### Co-occurring compensatory mutations

In addition, previous studies have indicated that compensatory mutations may influence the transmission and evolutionary success of MDR strains^9,35^. Therefore, we searched for co-occurring compensatory effects in samples resistant to both isoniazid and rifampicin. A total of 36 samples contained more than one ‘highly likely’ or ‘likely’ compensatory mutation. Such samples were found to have at least one mutation in *oxyR’-ahpC* and *rpoC* that occurred alongside one another. Co-occurring compensatory effects were primarily observed independently across sub-lineages. For example, the co-occurrence of *oxyR’-ahpC* c.-47G > GT was observed alongside *rpoC* I491V in lineage 4.2.2 only (Supplementary Figure S2). However, co-occurring putative compensatory mutations were also found across independent lineages, such as *oxyR’-ahpC* c.-48G > A and *rpoC* V483G which were observed in samples belonging to lineages 2, 4 and 6. This indicates that compensatory mutations may drive the evolution of MDR-TB either through convergent or strain-specific mechanisms.

### Protein structure analysis of potential compensatory mutations in *rpoA *and *rpoC*

**Figure 1:**
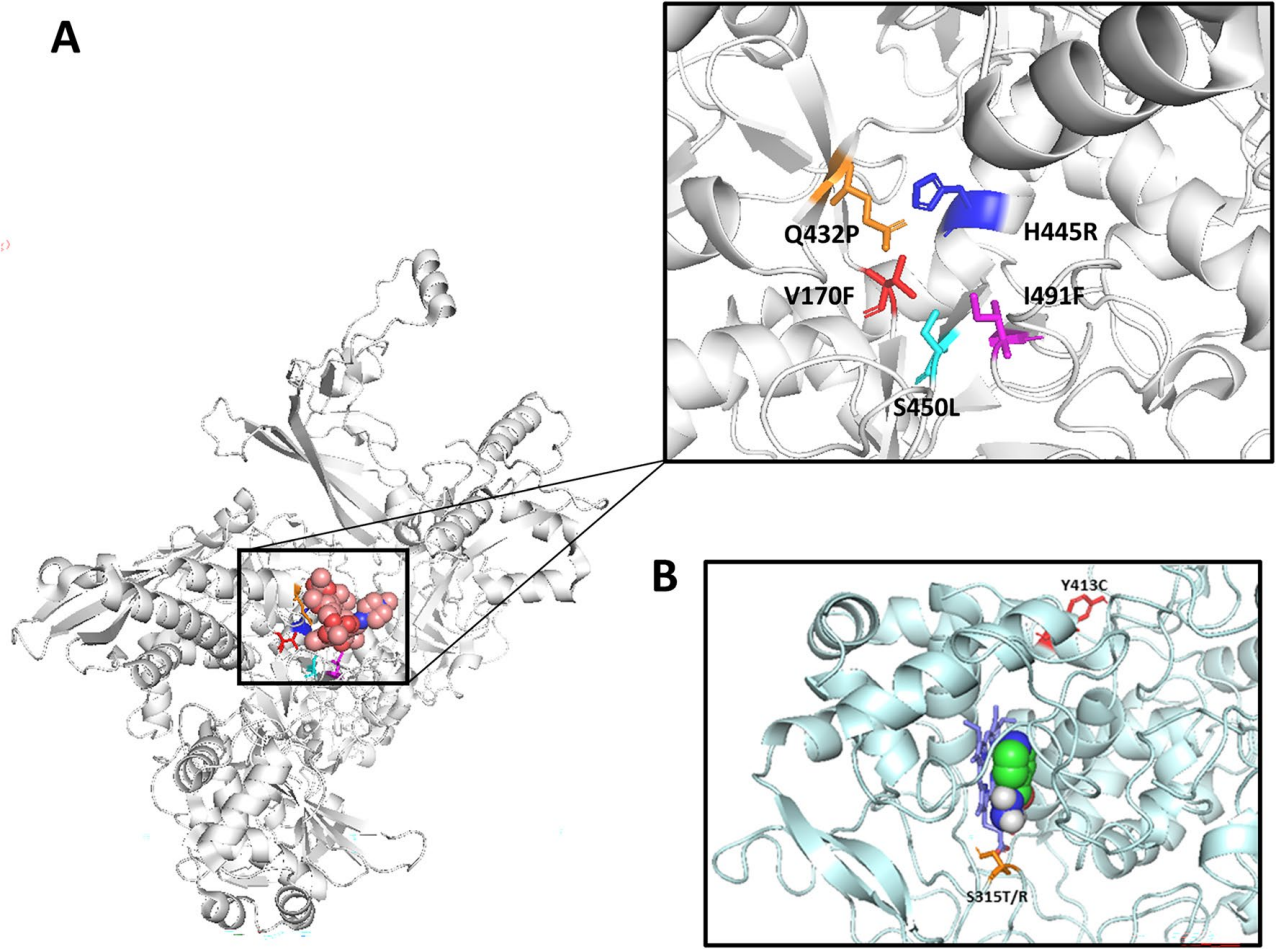
Drug-resistance mutations in the RNAP β-subunit (*rpoB*) and catalase peroxidase (*katG*). The RNAP β-subunit is shown in complex with the rifampicin ligand (spheres) (**A**). rpoB non-synonymous mutations are shown in the binding site regions as sticks and are coloured and labelled accordingly (**A**). Isoniazid (spheres) is shown interacting with the heme group of catalase peroxidase and is annotated with drug-resistance mutations (**B**).

To gain further insight into the mechanisms of the potential compensatory mutations identified using CompMut-TB, we assessed their impact on protein structure stability and protein–protein interactions (PPI). Most known drug-resistance mutations in *katG* and *rpoB* were predicted to have destabilising effects on either protein stability or PPI and were situated near to the drug binding pockets and ligand (Figure 1). Few drug-resistance mutations conferred stabilising effects on PPI, including *rpoB* Q432P and *katG* S315R, but showed mild destabilising effects on protein stability (Supplementary Table S4). The most prevalent drug-resistance mutations in the dataset were *rpoB* S450L and *katG* S315T that were predicted to have only mild effects on protein stability and PPI supporting previous results (Supplementary Table S4)^21^. Notably, putative compensatory mutations associated with *rpoB* S450L and *katG* S315T had smaller effect sizes, suggesting that the role of compensatory mutations may be reduced for drug-resistance mutations that have mild destabilising effects on protein structure and function (Supplementary Table S4). Most other drug-resistant mutations in *rpoB* and *katG* had moderate destabilising effects on protein structure, some of which were associated with compensatory mutations that had larger effect sizes (Supplementary Table S4).

**Figure 2:**
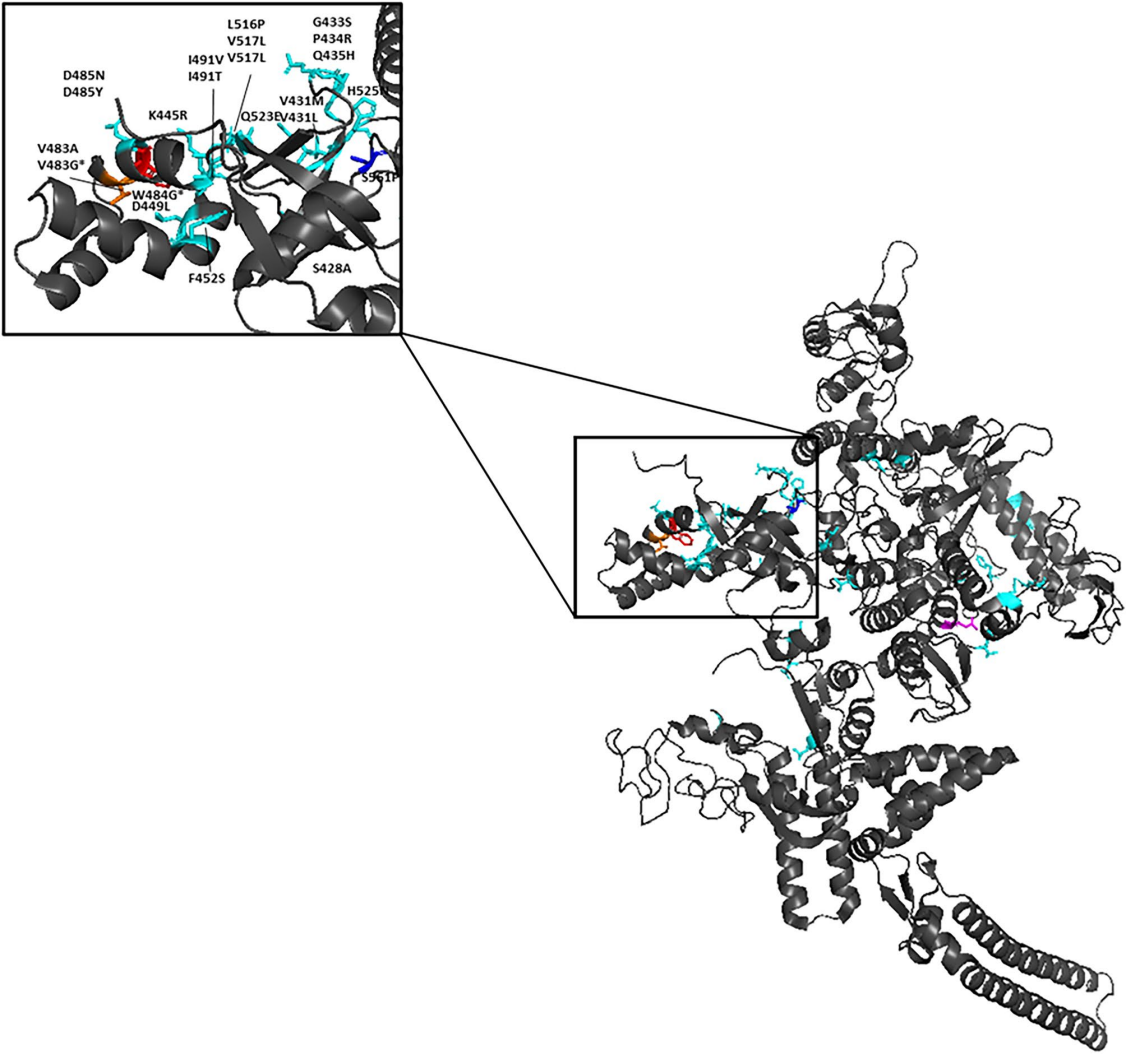
Putative compensatory mutations in the RNAP β’-subunit (*rpoC*). The RNAP β’-subunit is shown. Putative compensatory mutations in *rpoC* (identified in mediation and association analysis) are shown as sticks and are coloured according to their associated drug-resistance mutation in *rpoB* as shown in Fig. 1. The ‘zoomed’ in box shows a ‘hotspot’ region of mutations within the ‘arm’ of the β’-subunit. * indicates non-synonymous SNPs that mediated drug-resistance to more than one *rpoB* mutation.

**Figure 3:**
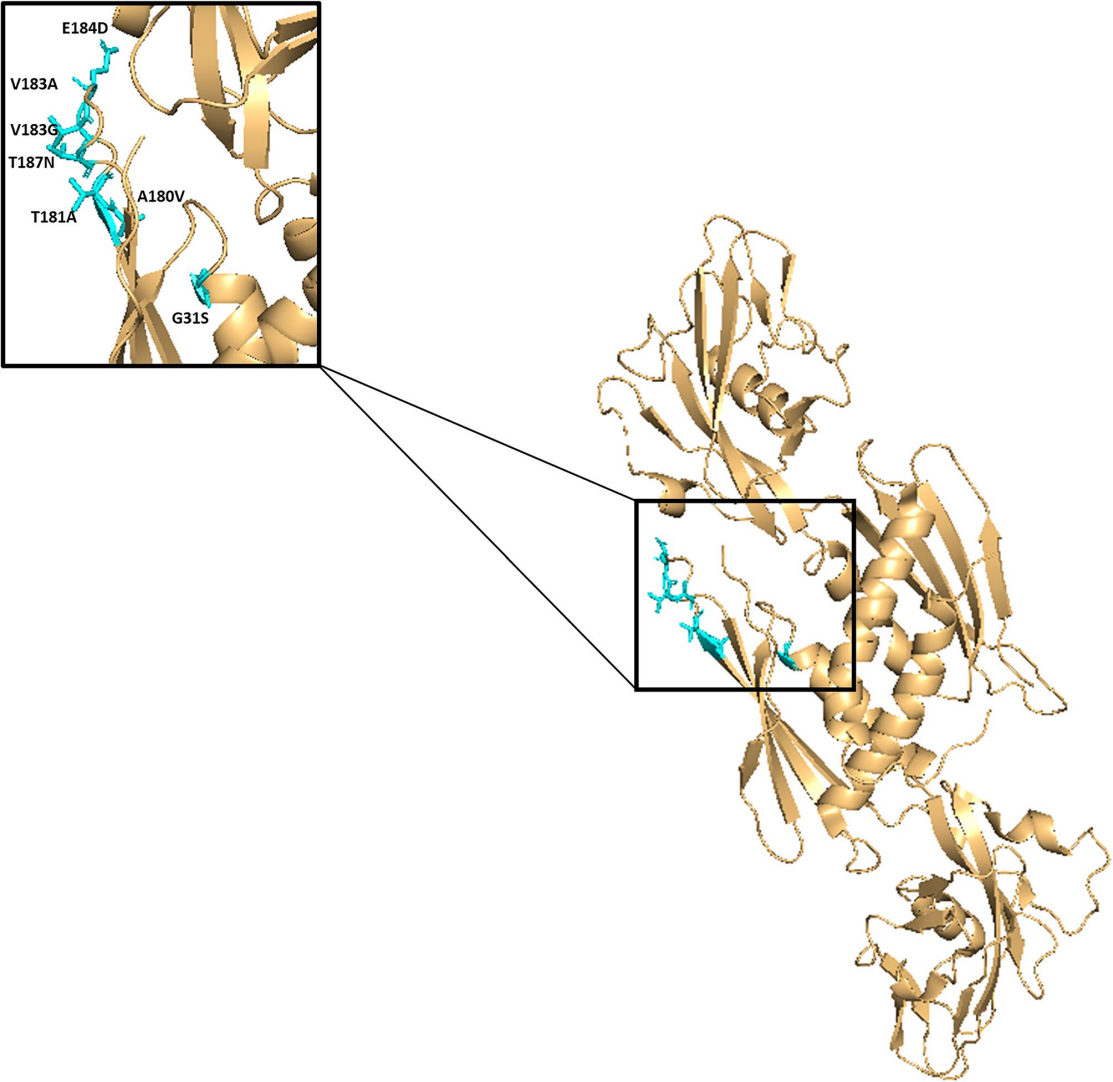
Putative compensatory mutations in the RNAP α-subunit (*rpoA*). The RNAP α-subunit is shown. Putative compensatory mutations in *rpoA* (identified in mediation and association analysis) are shown as sticks and are coloured according to their associated drug-resistance mutation in *rpoB* S450L as shown in Fig. 1. The ‘zoomed’ in box shows a ‘hotspot’ region of mutations in the α-subunit.

Most putative compensatory mutations in *rpoA* and *rpoC* were not located near to either *rpoB* mutations or rifampicin (> 20 Å), indicating that they may have indirect compensatory effects on protein structure (Supplementary Table S4). Many of the *rpoC* mutations were situated on the within the ψβ-barrel structure of RNA polymerase β’ subunit, whilst several are found at other sites (Fig. 2). Several mutations have previously been shown to interact directly with the RNAP β-subunit including V1252L/M, L507V, A734G, and S428A^39^. All *rpoA* mutations were located on the surface within a specific domain of the alpha subunit (Domain ID: e5uhcA1) and appear to interact with the β’-subunit (Fig. 3).

Most potential compensatory mutations in *rpoA* and *rpoC* had destabilising effects on protein stability and PPI. Several *rpoC* mutations had highly destabilising effects on protein stability (< -2 kcal/mol) and destabilising effects on PPI (0 to − 2 kcal/mol) (G433S, F452S, V483A, V483G, W484G, W484G, I491T and H525N), many of which are highly exposed residues (Supplementary Table S4). Meanwhile, six *rpoC* mutations (G332R, G332S, D485Y, D747A, Q1110H and Q1125H) and one *rpoA* mutation (T181A) were predicted to have stabilising effects (> 0 kcal/mol) on PPI (Supplementary Table S4). Furthermore, the combined impact of putative compensatory mutations and drug-resistance mutations was predicted using DynaMut2 [73]. The *rpoC* G332S, N416S and D485Y mutations, as well as *rpoA* G31S and T181A were predicted to stabilise RNA polymerase in the presence of *rpoB* S450L (Supplementary Table S4). Meanwhile, all other mutation pairs had a destabilising impact (Supplementary Table S4).

## Discussion

Compensatory mutations have been implicated in enhancing survival and transmission of multi-drug resistant TB strains^7,8,18,36^. Compensatory mutations are said to alleviate the fitness costs of drug-resistance mutations and have been described for several drugs, including isoniazid and rifampicin^5,7,37^. However, the extent of mutations that confer compensatory effects is unknown. Here, we developed CompMut-TB, a framework capable of identifying potential compensatory mutations. We leveraged large-scale WGS data to demonstrate the capability of CompMut-TB to identify potential compensatory mutations in genomic regions known to confer compensatory effects, such as *rpoA/ rpoC* (rifampicin) and *oxyR’-ahpC* (isoniazid).

CompMut-TB is dependent on both association and mediation analyses. Fisher’s exact tests are performed to screen mutations to find any mutations that are associated with a drug-resistant mutation and resistant phenotype. After screening, mediation analysis is used to explore the process by which a mediator variable (compensatory mutation) affects the relationship between the independent variable (drug-resistant mutation) and the outcome variable (drug-resistant phenotype), as determined by the significance of the indirect effect (*ab*). Whilst mediation analysis has been utilised in a diverse set of scientific disciplines including psychology, medicine, epidemiology, social sciences and biomedical research as a causal inference method, it is yet to be widely applied in a genomic context^38^. This has been driven in part by the large number of potential mediators in high-dimensional, genome-wide studies^39^. However, in this study we highlight that mediation analysis is a useful method for observing complex interactions between genomic variants that contribute towards a phenotype, such as the effect of compensatory mutations on drug-resistance. This was demonstrated by the identification of potential compensatory mutations in *rpoA* (N = 4)*, rpoC* (N = 47) and *oxyR’-ahpC* (N = 7)*,* many of which have previously been reported or validated. Therefore, mediation analysis may be applied to explore genotype–phenotype relationships in the future^40^. For example, mediation analysis may be utilised to explore epistatic interactions more widely by taking a multivariate analytical approach. The evolution of drug-resistance is not solely underpinned by drug-resistance mutations and compensatory mutations alone, but also the strain background and mutations associated with previous drug exposure. The association between compensatory mutations and lineage, such as for the Beijing lineage, has been explored previously, but could be enhanced further by searching for phylogenetic mutations that mediate or moderate drug-resistance^8^. In a similar vein, often patients receive primary treatment with a first-line drug such as isoniazid before the development of multi-drug resistance. Given that isoniazid-resistant genotypes increase the risk of developing drug-resistance to other drugs and share resistance mechanisms with ethionamide (e.g. in *inhA*), it is also plausible that they impact progression to further drug-resistance, such as pre-XDR and XDR phenotypes^41^. This suggests there is a complex network of epistatic interactions driving drug-resistance that could be explored further using mediation analysis.

To provide additional support that the framework could identify biologically meaningful compensatory mutations and drug-resistance mutations, we searched for experimental evidence in the literature that supported our results. Comas et al., (2012) identified 12 high-probability compensatory mutations from in vitro and in vivo strains that were considered to exhibit highly competitive fitness^5^. This included *rpoC* D485N and L516P that were identified within our study which are associated with strains that contain *rpoB* S450L. Further support for the compensatory effects of *rpoC* L516P was provided by showing this mutation can mitigate gene expression changes that occur due to *rpoB* S450L^42^. In addition, *rpoC* I491V has been shown to alter gene expression after rifampicin exposure, whilst isolates containing *rpoC* V483G have higher relative fitness than strains that only contain *rpoB* S450L^43^. Experimental evidence also provides support for three of the *oxyR’-ahpC* mutations identified. Earlier studies demonstrated that *oxyR’-ahpC* mutations driving AhpC overexpression were observed in KatG-deficient strains^11^. *M. smegmatis* has reduced INH sensitivity linked to o*xyR’-ahpC* mutations. The induction of such *oxyR’-ahpC* mutations in wild-type *M. tuberculosis* has confirmed their role in the activation of AhpC expression, which contributes to the fitness of INH resistant strains^6,11,14^. More specifically, isolates with *oxyR’-ahpC* mutations including -48G > A and -52C > T have shown to have higher average MIC than isolates with no *oxyR’-ahpC* mutations^44^. Overall, this highlights the ability of our framework to identify compensatory mutations that have previously undergone experimental validation.

Furthermore, CompMut-TB also identified mutations that have not been previously reported as a compensatory mutation, including *rpoC* L566V, A734G, V1039G, P1040T and *ahpC* -47G > GT. This outcome suggests that CompMut-TB can be used to identify new potential drug-resistance variants, which could be implemented in mutation catalogues to support profiling drug-resistant strains^45^. However, this would be dependent on the validation of the fitness impact of potential compensatory mutations.

Notably, none of the potential compensatory mutations in this study, including those that have been previously reported, are currently implemented in the WHO drug-resistance mutation catalogue^33^. Despite WHO endorsement of the Cepheid Xpert® MTB/XDR and targeted next generation sequencing assays, which in part depend on *ahpC* promoter mutations to classify INH resistance, the WHO catalogue was developed using the SOLO association algorithm that cannot intrinsically classify compensatory mutations^34^. Therefore, all putative compensatory mutations are assigned ‘uncertain significance’ within the catalogue and are not used for resistance-profiling. It is acknowledged that other methods are required to identify compensatory mutations and mutations with low positive predictive value, such as regression-based approaches^34^. This highlights the value of developing a robust statistical framework to identify compensatory mutations as presented by our study. Whilst compensatory mutations are not considered as directly causative of drug-resistance, their inclusion may help to boost the sensitivity of genetic-based DSTs. Rarer mutations may not be detected through association or systematic analysis and, therefore, will not be included in mutation catalogues. In such cases, compensatory mutations that co-occur with rare drug-resistance mutations can act as a representative to enhance predictive performance, as suggested for rare *katG* mutations associated with mutations in the *ahpC* promoter region^46^.

We also assessed the impact of potential compensatory mutations on protein stability and identified their location within the protein structure. Most *rpoC* mutations were found in the ψβ-barrel structure of the RNA polymerase β’ subunit, a region that appears to be a ‘hotspot’ for compensatory mutations within our and previous studies^26,47^. Likewise, we identified a ‘hotspot’ region for putative compensatory mutations in *rpoA* that interacts with the RNA polymerase β’-subunit. Mutations in *rpoA* and *rpoC* mutations may restore the function of RNA polymerase through conferring stabilising or destabilising effects on protein stability or PPI in a complex manner and there may be other mechanisms by which missense mutations contribute to protein stability^48^. For example, Portelli et al., showed that compensatory mutation *rpoC* V483G cancelled out the change in accessible surface area (ASA) for *rpoB* S450L. In addition, the impact of compensatory mutations on bacterial fitness and survival are likely to differ between strains in neutral conditions and in the presence of a drug^48^. Whilst this provided an indication of the functional effects of missense mutations, ΔΔG is not a direct measure of the impact on fitness and further experimental validation is required.

Finally, we acknowledge that there are limitations of this study that should be addressed in future. Firstly, we used previously published data for the analysis that have been collected using a convenience sampling approach and only focussed on SNPs. This is likely to have affected the power of our framework to detect rare putative compensatory mutations (including Indels) that have indirect effects on drug-resistance, and we were also unable to look at temporal trends or direct effects on transmission or growth. Whilst all pDST data was obtained using robust, WHO approved methods, the use of different assays could affect resistance scoring. A quantitative analysis using minimum inhibitory concentrations (MIC) could improve on this in the future to observe the cumulative effects of mutations on MIC^49^. Additionally, the distribution of non-synonymous SNPs in *rpoABC* may not be representative of the global population, especially in lineages 5, 6 and 7. However, we only considered mutations that were present in more than one lineage. Whilst we chose to do this to prevent confounding from population structure, which is essential for mediation analysis, previous studies have reported putative compensatory mutations that belong to single lineages^8,9,24,26^. Notably, the co-occurrent resistant patterns of compensatory mutations were observed in individual sub-lineages, further suggesting their role in lineage-specific MDR transmission^9,35^. Therefore, further research should be carried out to identify lineage-specific compensatory mechanisms as strain genetic backgrounds are known to play a role in the evolution of drug-resistance mechanisms^50^. Also, compensatory mutations have previously been detected in heteroresistant samples which were masked in this analysis due to their low frequency^21^. Consequently, the role of compensatory mutation in heteroresistance merits further exploration. In addition, we only considered putative compensatory mutations in *oxyR’-ahpC*, *rpoC* and *rpoA*. Mutations outside of the RRDR in *rpoB* have also been previously reported to have compensatory effects on rifampicin-resistance and therefore other genomic regions should be considered^23,36^. Additionally, mediation analysis could be utilised for the exploration of potential compensatory mutations in other bacterial species, such as *Escherichia coli* and *Salmonella typhimurium* that have previously shown to exhibit similar fitness costs and compensation^51,52^.

## Methods

### Phenotypic and sequencing data

The final dataset is comprised of genotypic and phenotypic information for 18,396 *Mycobacterium tuberculosis* isolates that had previously been curated from the public domain^53,54^. All original samples (*n* = 38,433) had undergone whole genome sequencing (WGS) and phenotypic drug susceptibility tests (pDSTs) as described previously^17^. Raw reads were aligned to the H37Rv reference genome (NC_000962.3) and single nucleotide polymorphisms (SNPs), as well as insertions and deletions (Indels), were called using SAMtools (BCFtools v1.9) and GATK (v4.1.6) software^55,56^. To be included in the final analysis, all samples had at least 10 × coverage across 90% of the genome and had adequate coverage across all genomic regions of interest (median coverage: *ahpC* 92.8; *katG* 85.8*; rpoA* 87.8; *rpoB*: 89.4; *rpoC* 90.5). A minor number of calls were heteroresistant and therefore were masked in the analysis (*n* = 28). Genotype calls with less than 70% read support were marked as missing and sites were excluded if greater than 10% of data was missing or if the site was monomorphic. Missing genotypic information was imputed by the most frequent allele. The pDSTs followed WHO recommended protocols (see^53^). For most isolates, the pDST data was incomplete and isolates with missing related data for rifampicin or isoniazid were excluded from the respective analyses, leaving 18,396 samples with pDST data for at least one drug.

### Association analysis

Before undergoing statistical analysis, mutations were extracted from the dataset using gene boundaries obtained from the MycoBrowser database^57^. Lineage informative mutations were excluded from the analysis to improve the detection of putative compensatory mutations and prevent confounding from population structure. Only missense mutations in *rpoA, rpoB, rpoC,* and *katG* were included in the analysis as they were considered more likely to play a role in drug resistance. Two stages of association analysis were carried out. Firstly, Fisher’s exact tests were performed between mutations in *rpoB* and *rpoC* or *rpoA,* as well as *katG* and *oxyR’-ahpC* to identify pairs of mutations within these genes that are associated with one another. Secondly, Fisher’s exact tests were conducted to identify mutations that were associated with rifampicin (*rpoA, rpoB,* and *rpoC*) and isoniazid (*katG* and *oxyR’-ahpC*) resistance. *P*-values were adjusted for multiple testing using the Benjamini–Hochberg procedure. SNP-pairs were prioritised if both SNPs were associated with rifampicin/isoniazid resistance and one another (*P-*value < 0.05).

### Mediation analysis

To explore the effects conferred by potential compensatory mutations, mediation analysis was conducted using the structure equation modelling package *lavaan* (v0.6–10)^58^*.* In all mediation analyses, non-synonymous mutations in *rpoB* or *katG* were input as the exogenous/binary independent variable (X) [reference allele = 0, alternative allele = 1] and pDST results (isoniazid/rifampicin) were entered as the endogenous/binary dependent variable (Y) [resistant = 1, susceptible = 0]. Non-synonymous SNPs in either *rpoC* or *rpoA* (*rpoB*) and intergenic mutations in *oxyR’-ahpC* (*katG*) were included as the mediators in the analysis (M). To perform mediation analysis a probit regression model approach was taken using the DWLS estimator and bootstrapping procedure (1000 bootstrapped samples). To determine a causal relationship from mediation analysis it is assumed that there is no confounder affecting the relation between the endogenous, exogenous, and mediator variables. Therefore, phylogenetically informative SNPs were removed from the analysis. Perfect multicollinear SNPs (correlation coefficient equal to 1) were also removed to avoid yielding results with biased path coefficients. The direct effect between *rpoB* or *katG* mutations and rifampicin resistance were estimated by the *c* coefficient. Mediation by mutations was measured by the indirect effect coefficient (*ab*).

To identify putative compensatory mutations, we identified SNPs in *rpoC*, *rpoA* and *oxyR’-ahpC* that conferred complementary mediation, where the direct (*c*) and indirect effects (*ab*) were both significant (confidence intervals did not contain 0). In such cases, there is a significant relationship between SNPs in *rpoB* and *rpoC/rpoA* or *katG* and *oxyR’-ahpC,* as well as a direct relationship between drug-resistance mutations and the resistant phenotype. Examples of indirect-only mediation and competitive mediation were also recorded. Indirect-only (full) mediation occurred if the outcome had a significant indirect effect coefficient (*ab*) but had non-significant direct effect (*c*), whilst competitive mediation was indicated by significant direct and indirect estimates that pointed in opposite directions.

### Identifying putative compensatory mutations

We combined the results of the mediation and association analyses to identify putative compensatory mutations. Mutations were grouped into categories that described how likely they were to play a compensatory role and were assigned according to statistical and practical evidence. We hypothesized that mutations were highly likely to be a compensatory mutation if (i) they were associated with a potential drug-resistance mutation and resistant phenotype, (ii) they displayed significant complementary mediation between the drug-resistance mutation and resistant phenotype, and (iii) if the standardised indirect effect contributed to greater than 5% of the standardised total effect (*ab*/total). Mutations were considered ‘likely’ if they satisfied the first two criteria but contributed < 5% to the total effect and ‘highly unlikely’ if they did not satisfy criterion (i). All other mutations, including those that displayed competitive and full mediation, were considered ‘unlikely’ and were either insignificant or the confidence intervals of direct and indirect effects covered zero. However, full mediators were also screened to identify any drug-resistance mutations that required the presence of a compensatory mutation to bring about drug-resistance. Under conditions with high selective pressure, such as high drug concentration, it is also plausible that mutations are important for the survival of the organism. To ensure that all putative compensatory mutations have compensatory effects, we checked that all mutations only occurred in the presence of a mutation in the drug-resistance gene and that they did not occur independently. An overview of the systematic framework is available (Supplementary Figure S3). All mutations were cross referenced to mutations in the TB-Profiler database, as well as those previously reported in the literature to identify unknown potential compensatory mutations^45^.

### Predicting the effects of mutations on protein structure

All putative compensatory mutations were mapped onto the crystal structure of RNA polymerase (PDB ID:5UHA), as well as in complex with rifampicin (PDB ID:5UHC) and catalase peroxidase (PDB ID:1SJ2). Docking was performed using AutoDock Vina (v1.1.2) to predict the orientation and position of *katG* in complex with isoniazid (PubChem CID: 3767). Each single missense mutation was subject to graph-based signature algorithms mCSM-stability (protein structure stability) and mCSM-PPI (protein–protein interface stability) using the *mCSM* webservers (http://biosig.unimelb.edu.au/mcsm/)^59,60^. To investigate the effects of multiple mutations on protein stability, pairs of compensatory and drug-resistance mutations were also input to the DynaMut2 web-server (http://biosig.unimelb.edu.au/dynamut2)^61^. SNPs were classified as highly destabilising, mildly destabilising, mildly stabilising, moderately stabilising, or highly stabilising according to the default ∆∆G thresholds. Protein structures were visualised using PyMol (v2.4.1)^62^.

### Implementation

All statistical analyses were carried out using R software (v3.6.1). The code for CompMut-TB can be found in a dedicated GitHub repository (https://github.com/NinaMercedes/CompMutTB)) and can be run as a command-line tool from FASTQ, VCF or genotype matrices.

### Supplementary Information

Supplementary Information 1.

Supplementary Information 2.

Supplementary Information 3.

Supplementary Information 4.

Supplementary Information 5.

## Data Availability

Datasets used to generate the results of this article are available in the NCBI repository. No new isolates were sequenced during this study. A list of sample and project accession numbers are available in the GitHub repository: https://github.com/NinaMercedes/CompMutTB.
