# Supplementary material for: Large-scale statistical analysis of Mycobacterium tuberculosis genome sequences identifies compensatory mutations associated with multi-drug resistance
Source: Sci Rep. 2024 May 29;14:12312. doi: 10.1038/s41598-024-62946-8 (PMC11137121; doi:10.1038/s41598-024-62946-8)
Supplement: Supplementary file 1 — Supplementary Information 1. [file 41598_2024_62946_MOESM1_ESM.pdf]

Large-scale statistical analysis of *Mycobacterium tuberculosis* genome sequences identifies compensatory mutations associated with multi-drug resistance

Nina Billows[1,2], **Jody Phelan**[2], **Dong Xia**[1], **Yonghong Peng**[3], **Taane Clark**[2,4], **Yu-Mei Chang**[1]

[1]Royal Veterinary College, University of London, London, UK

[2]Faculty of Infectious and Tropical Diseases, London School of Hygiene & Tropical Medicine, London, UK

[3]Manchester Metropolitan University, Manchester, UK

[4]Faculty of Epidemiology and Population Health, London School of Hygiene & Tropical Medicine, London, UK

*Nina.Billows@LSHTM.ac.uk

Supplementary Figures

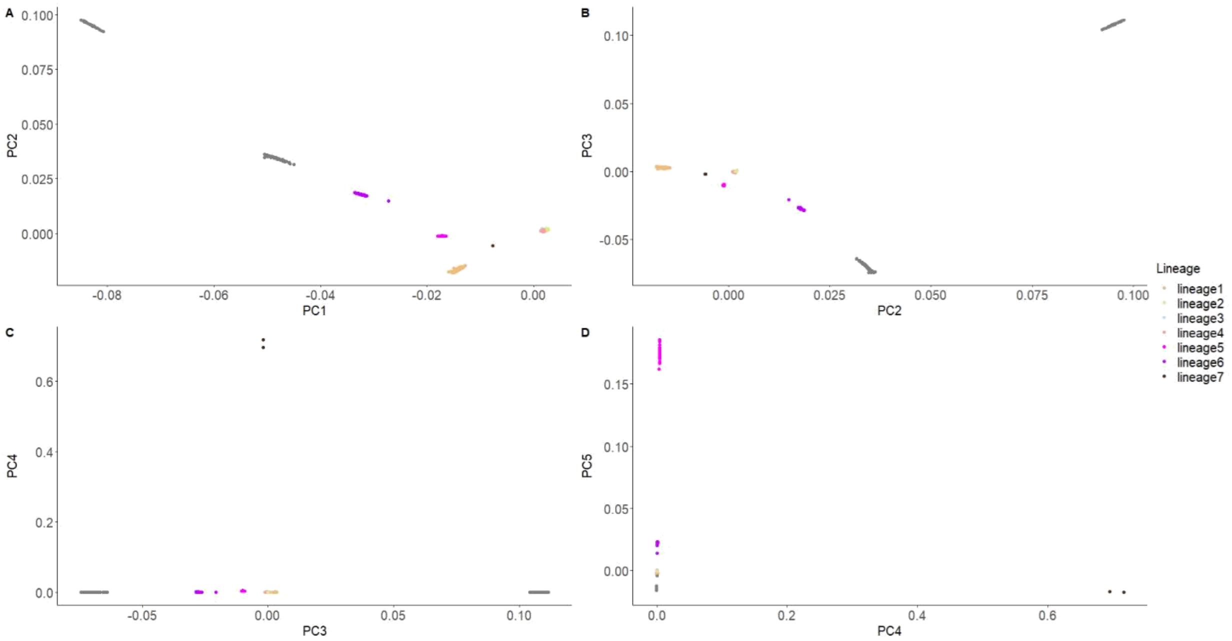

Supplementary Figure S1. Principal Component Analysis of the global *M. tuberculosis* dataset. Principal components (PC) 1-5 are plotted for 18,396 isolates in the dataset and are colour coded by lineage. PCA was carried out using PLINK (v1.90) software.

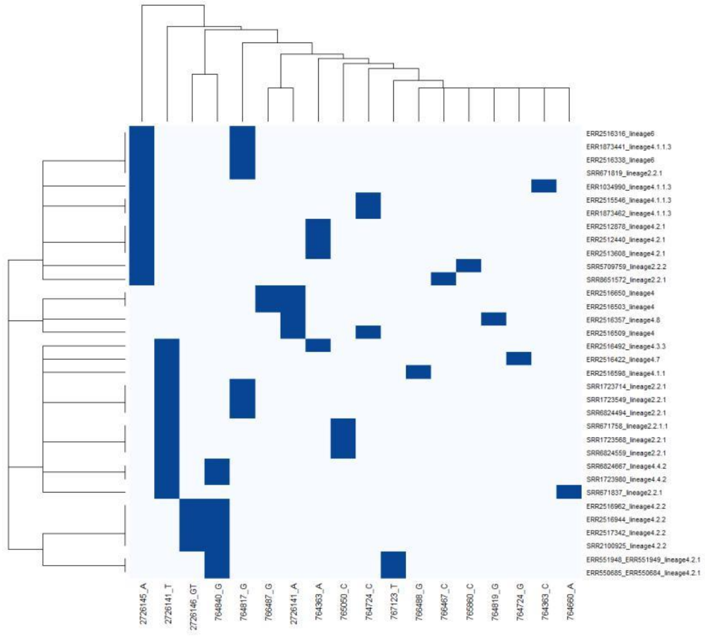

Supplementary Figure S2. Co-occurring putative compensatory mutations in MDR isolates.
MDR isolates with more than one putative compensatory mutation are shown alongside their respective mutations. The lineage of each sample is also shown.

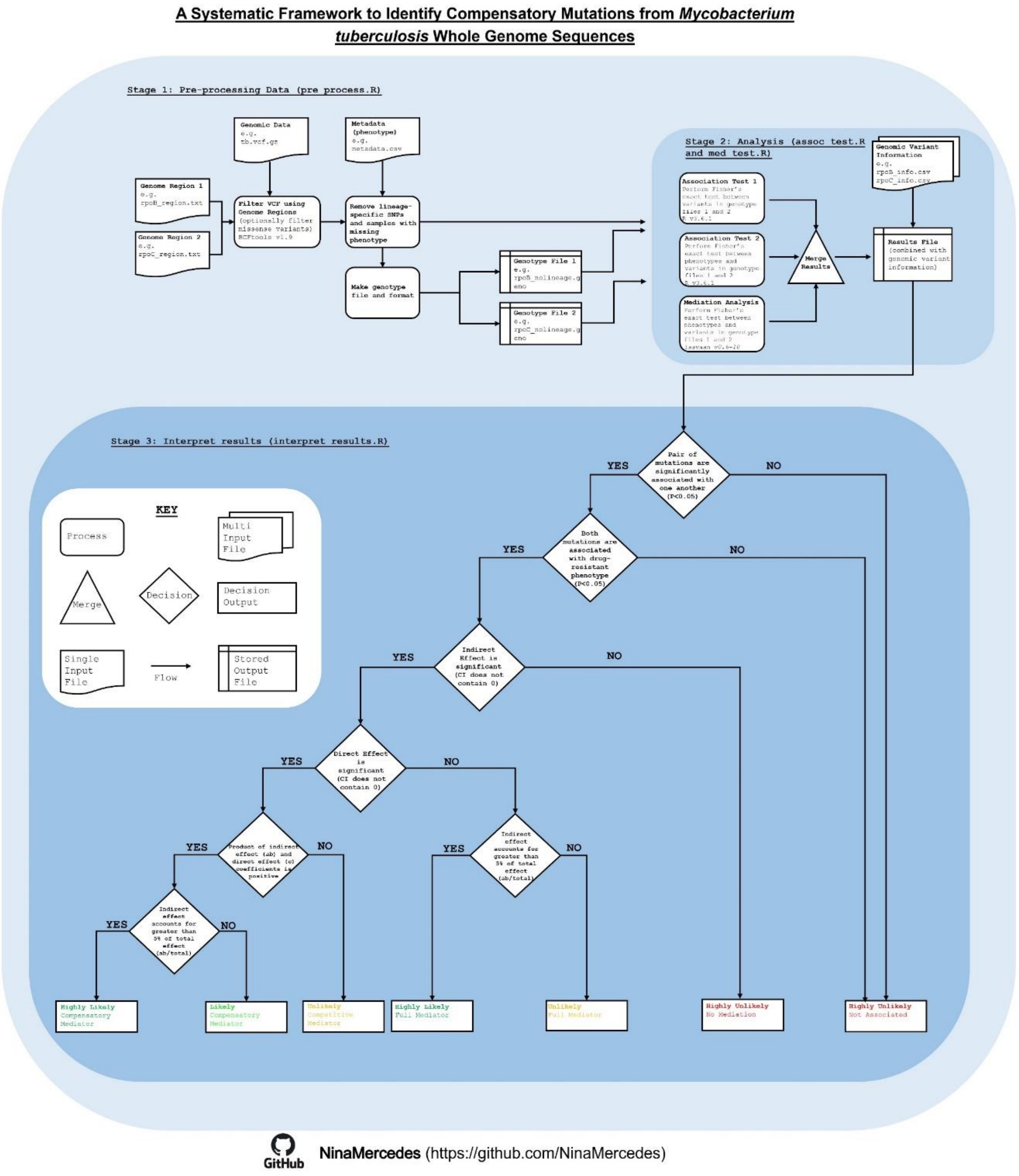

Supplementary Figure S3. Overview of systematic framework to identify compensatory mutations.
Flow diagram summarises three stages of analysis and the key is shown.
